# Exploring the Mediation Effect of Metabolite Levels on the Association Between Gut Microbiota and HCC: A two-step, two-sample bidirectional Mendelian Randomization

**DOI:** 10.7150/jca.96579

**Published:** 2024-05-30

**Authors:** Bingchen Xu, Lianxin Zhu, Pan Hu, Wang Yao, Miaola Ke, Zhihua Zhu

**Affiliations:** 1 State Key Laboratory of Oncology in South China, Sun Yat-sen University Cancer Center, Department of Minimally Invasive Intervention, Guangdong Provincial Clinical Research Center for Cancer, Collaborative Innovation Center for Cancer Medicine, Guangzhou 510060, P. R. China.; 2 Medical College of Nanchang University, Nanchang 330088, P.R. China; Queen Mary University of London, London, United Kingdom.; 3 The First Affiliated Hospital of Sun Yat-sen University, Department of Interventional Oncology, Guangzhou 510080, P.R. China.; 4 State Key Laboratory of Oncology in South China, Sun Yat-sen University Cancer Center, Department of Blood Transfusion, Guangdong Provincial Clinical Research Center for Cancer, Guangzhou 510080, P.R. China.; 5 State Key Laboratory of Oncology in South China, Sun Yat-sen University Cancer Center, Department of Thoracic Surgery, Guangdong Provincial Clinical Research Center for Cancer, Collaborative Innovation Center for Cancer Medicine, Guangzhou 510060, P. R. China.

**Keywords:** Gut Microbiota, Hepatocellular Carcinoma, Phosphoethanolamine, Mendelian Randomization, *Clostridium leptum*

## Abstract

**Background**: Although the gut microbiota is one of the risk factors for liver cancer, it remains unclear whether the level of metabolites mediates this association.

**Methods**: Utilizing summary data from genome-wide association studies (GWAS), we conducted a two-sample Mendelian Randomization (MR) analysis to explore the causal links between GM, metabolites, and HCC. A two-step MR analysis quantitatively assessed the effect of metabolite-mediated GM on HCC.

**Results**: In our study, we demonstrated that *Clostridium leptum* was identified as a protective factor against HCC, with no evidence of reverse causality (Inverse-variance weighted [IVW], OR: 0.62 [95% CI, 0.42-0.91]; p = 0.016). Our study also found that the potential connection between the GM and HCC may be mediated by the level of metabolites. An increase of one standard deviation in *C. leptum* abundance led to a 38% decrease in HCC risk (OR: 0.62 [95% CI, 0.42-0.91]), with a 9% reduction in phosphoethanolamine (PE) levels (OR: 0.91 [95% CI: 0.84-0.99]). PE's mediation proportion was established as -6.725% (95% CI, 12.96% to -26.41%).

**Conclusion**: Our results demonstrate that increasing specific GM abundance can lower HCC risk, mediated by PE levels. We offer new prevention and treatment targets for HCC by adjusting GM.

## 1. Introduction

According to GLOBOCAN 2020 data, liver cancer ranks sixth in terms of malignant tumor incidence and third in mortality rates 1. With a continuous rise in liver cancer incidence, its global burden is substantial. By 2040, there will be 1.4 million new cases of liver cancer, resulting in 1.3 million deaths globally 2. Late-stage diagnosis, postoperative recurrence, and limited treatment options contribute significantly to the poor prognosis of liver cancer patients. Therefore, researching early intervention and prevention strategies for liver cancer is crucial to mitigate the societal health burden 3, 4.

Extensive research had revealed the imbalance of gut microbiota can lead to the occurrence of liver cancer, as the gut microbiota and their metabolites can influence the liver environment via the portal vein system 5. Grat *et al.* 6 found that the abundance of *Escherichia coli* in the feces of HCC patients was significantly higher than that in the feces of healthy controls. Meanwhile, some Mendelian randomization studies have also provided evidence of a causal relationship between gut microbiota and hepatocellular carcinoma (HCC) 7, 8.

Metabolites, as intermediate or end products of metabolic reactions, are influenced by various factors such as genetics, diet, and GM 9, 10. Conversely, they can also influence the development of HCC 11. Chen *et al.* found gut flora disequilibrium promotes liver cancer initiation by modulating tryptophan metabolism and up-regulating elevated sterol regulatory element-binding protein 2 12. Research by Greten and colleagues established that the GM controlled anti-tumor immunity in liver cancer by modulating bile acid metabolism 13.

These studies suggest that the GM may influence the development of HCC, and that the level of metabolites can also impact HCC. However, there have been no studies exploring the causal relationship between gut microbiota, HCC, and metabolites currently. Therefore, determining whether the levels of metabolites interact with the GM in the process of HCC occurrence is crucial. To ensure causality between these three factors, in this study, we utilized the data from the latest, large-scale genome-wide association study (GWAS) for Mendelian randomization (MR) analysis 14-16.

MR is a powerful method for causal inference in which randomly allocated genetic variants are used as instrumental variables (IV) for a phenotype 17. Given the random allocation of genetic variants at conception, MR can be used to overcome some of the biases inherent in causal inference in other analyses. For example, estimates are not biased owing to unmeasured confounding between an exposure, mediator, or outcome 18. The primary objective of this study is to explore the causal relationship between GM and HCC, as well as the regulatory role of metabolites. We aim to translate clinically relevant findings related to the GM, holding significant implications for the prevention and treatment of liver cancer.

## 2. Methods

### 2.1 Study design

Genetic variants serving as effective instrumental variables (IVs) must satisfy three core assumptions: i) the relevance assumption—the selected IV directly correlates with the exposure; ii) the independence assumption—the chosen IV is unrelated to any confounding variables between the exposure and the outcome; and iii) the exclusion restriction assumption—the selected IV exclusively influences the occurrence of the outcome through the exposure and not through alternative pathways.

Mediation analysis typically estimates three parameters: i) the total effect (the effect of the exposure on the outcome through all potential pathways); ii) the direct effect (the remaining effect of the exposure on the outcome that operates through pathways other than the specified mediator or set of mediators); and iii) the indirect effect, also known as the mediating effect (the path from exposure to outcome that operates through the mediators) 18-20.

**Figure 1:**
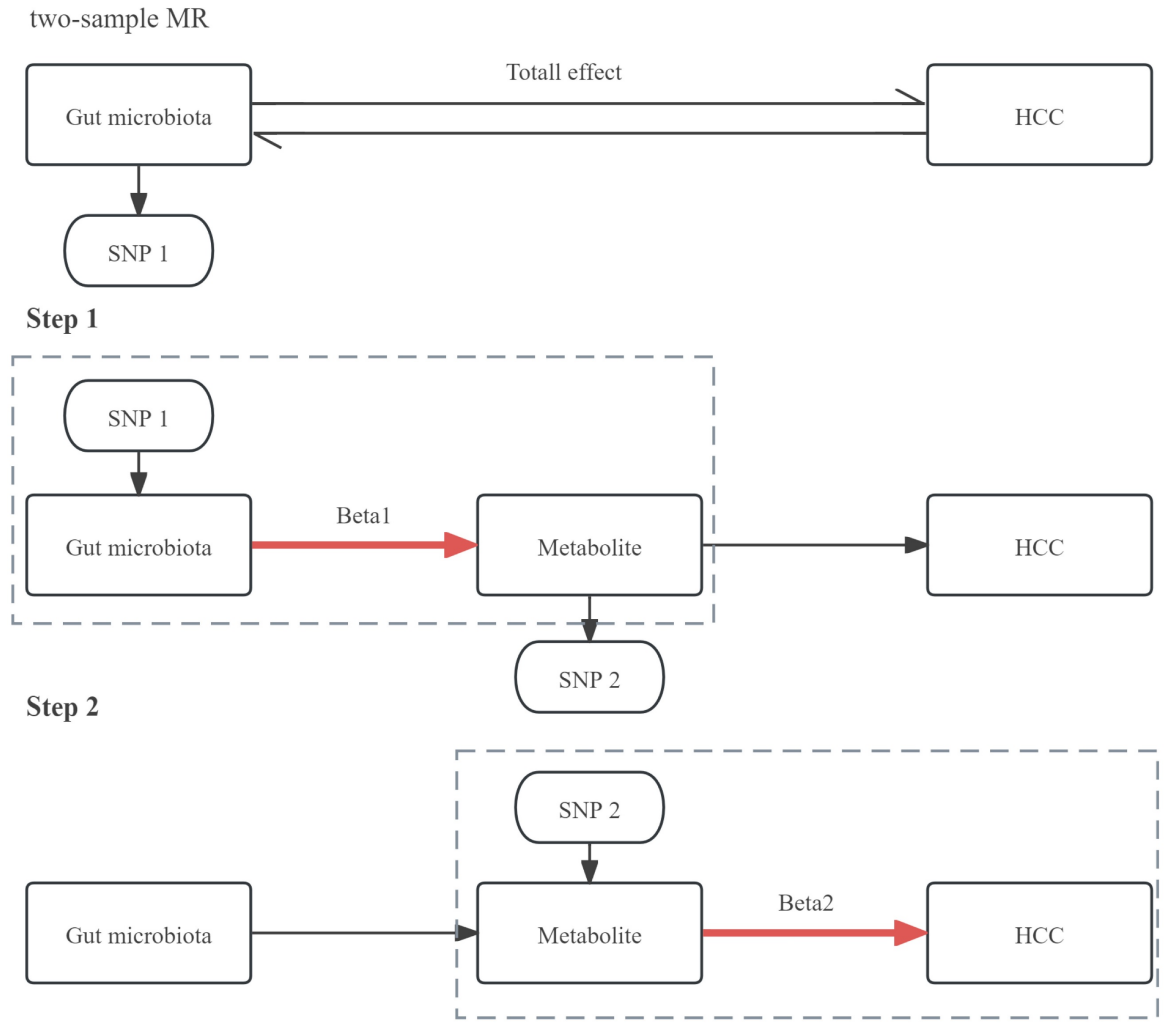
The design of a two-step MR analysis between GM and HCC mediated by plasma metabolites. SNP1: GM that is highly related to the risk of HCC; SNP2: metabolite that is associated with the GM; HCC: hepatocellular carcinoma.

First, we employed a two-sample bidirectional MR analysis to determine the causal relationship between GM and HCC, identifying GM highly associated with HCC risk as *SNP1* and obtaining *Total effect*. Next, we conducted a two-step MR for mediation analysis. The first step involved a two-sample MR between the selected gut microbiota *SNP1* and plasma metabolites, screening for metabolites associated with GM as *SNP2* and obtaining *Beta1*. The second step involved a two-sample MR between the selected metabolite *SNP2* and HCC, resulting in *Beta2*. The study design was illustrated in Figure 1.

### 2.2 GWAS summary data sources

The GWAS data for GM were obtained from the Dutch Microbiome Project (DMP), published in 2022^14^. The study utilized shotgun metagenomic sequencing on fecal samples from 7,738 individuals enrolled in the DMP, matching their imputed genotypes to differences in taxa and pathway abundances. This effort identified 207 taxa and 205 GM metabolic pathways, representing the composition and function of the GM. All volunteers were from the northern Netherlands.

The GWAS data for metabolites were sourced from a study published by Chen *et al.* in 2023. The researchers conducted GWAS on 1,091 blood metabolites and 309 metabolite ratios in 8,299 individuals from the Canadian Longitudinal Study on Aging (CLSA) cohort 15. This investigation revealed associations with 690 metabolites at 248 loci and associations with 143 metabolite ratios at 69 loci.

The outcome variable of HCC was obtained from GWAS summary data of the latest R10 version released by the FinnGen consortium on December 18, 2023. This dataset comprises 500 cases, 314,193 controls, and 21,303,829 single-nucleotide polymorphisms (SNPs). The FinnGen study is a large-scale genomics initiative that has analyzed over 500,000 Finnish biobank samples, correlating genetic variation with health data to understand disease mechanisms and predispositions 16.

Detailed information for the three databases is provided in Table 1. This study utilized publicly available published GWAS data, with all informed consent and ethical approvals previously obtained in the original studies. Therefore, no additional ethics approval or consent to participate was deemed necessary for this study.

### 2.3 Two-step MR analysis

#### 2.3.1 Selection criteria for IVs

The criteria for selecting instrumental variables are as follows. (1) We chose independent genetic variants at the genome-wide significant level (p < 1×10^-5^) as instruments in our MR analyses. Additionally, we included all SNPs using a less stringent cut-off in our MR analyses to augment the number of SNPs available for sensitivity analyses. This approach aimed to enhance robustness 21, 22. (2) Linkage disequilibrium (LD) between SNPs was assessed using the 1,000 Genomes Project European Sample data as a reference panel 23. SNPs with an LD (r^2^) > 0.001 or LD < 1,000 kb were excluded. (3) The F-statistic was employed to assess the strength of instrumental variables. We excluded genetic variants with an F-statistic < 10 to mitigate bias introduced by weak instrumental variables. The formula for F is F = Beta^2^/SE^2^. (4) Liver cirrhosis, hepatitis B virus infection, and other factors are risk factors for HCC and could potentially act as confounding variables. Therefore, we utilized the PhenoScanner V2 database to identify and exclude instrumental variables directly related to hepatitis and liver cirrhosis.

#### 2.3.2 Outcome variable selection criteria

The outcome variable was obtained by matching the corresponding instrumental variables in a database that includes the outcome variable.

#### 2.3.3 Main analysis methods

The two-sample MR analysis employed various statistical methods to evaluate the causal relationship, including MR-Egger, Weighted Median (WM), Inverse-Variance Weighted (IVW), Simple Mode, and Bayesian weighted. The IVW method was designated as the primary MR analysis technique 24. WM provides more reliable causal effect estimates when effective instruments are lacking 25. A Bayesian approach allows incorporation of current knowledge into the analysis via informative prior distributions, minimizing the impact of confounding factors on the results to the greatest extent possible 26.

#### 2.3.4 Sensitivity analysis

To mitigate the potential impact of underlying pleiotropy on the final results, we employed various sensitivity analysis methods. (1) Cochran's Q test was utilized to examine heterogeneity among selected SNPs. If significant heterogeneity is present (p < 0.05), the random-effects IVW method will be chosen; otherwise, the fixed-effects IVW method will be employed 27, 28. (2) Egger regression assessed whether multiple instrumental variables exhibit horizontal pleiotropy. The intercept represents the average pleiotropic effect across the genetic variants. A p-value of the intercept less than 0.05 (p < 0.05) and a large distance from zero indicate potential horizontal pleiotropy 29. (3) The MR pleiotropy residual sum and outlier (MR-PRESSO) test identified horizontal pleiotropic outliers in multi-instrument summary-level MR testing to eliminate outlier SNPs and estimate corrected results 30. A p-value less than 0.05 (p < 0.05) indicated potential horizontal pleiotropy.

#### 2.3.5 Visualization of results

To enhance the interpretability of MR analysis results, various visualizations were created 31. A leave-one-out analysis systematically removed one SNP at a time to assess overall analysis robustness. Scatter plots illustrated the impact of each SNP on exposure and outcome, capturing the collective trend. Forest plots, utilizing the Wald ratio method, elucidated individual instrumental variables' contribution to the overall causal estimate. Funnel plots helped to visualize potential bias in SNP selection.

### 2.4 Bidirectional MR analysis

We conducted reverse MR analysis between GM, metabolites, and HCC to confirm the absence of reverse association among them. This ensured that the mediation pathway could only proceed in the direction of exposure → mediator → outcome, as illustrated in Figure 1. The remaining analytical steps are analogous to the forward MR analysis described earlier. All MR analyses were performed using R version 4.2.0 (https://www.r-project.org/) with the “TwoSampleMR,” “MR-PRESSO,” and “ggplot2” packages.

### 2.5 Mediation analysis

Furthermore, we conducted a two-step MR analysis employing mediation analysis to explore whether metabolites mediated the causal pathway from GM to HCC. The total effect was decomposed into a mediating effect and a direct effect. The formula utilized for calculating the direct effect is: Direct Effect=Total Effect-Mediating Effect 19.

**Figure 2:**
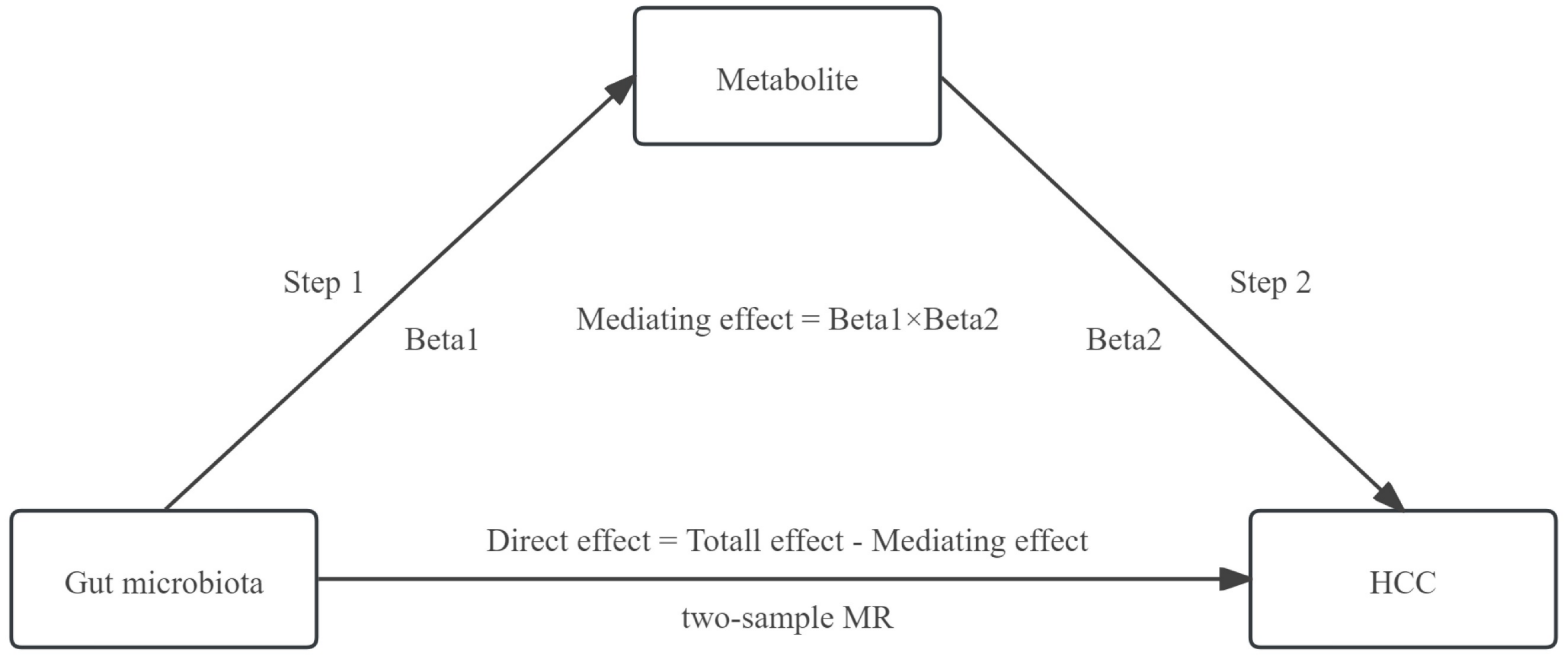
The steps of the mediation analysis.

The mediating effect of GM on HCC was then decomposed into i) the causal effect of the exposure on the mediator (beta1) and ii) the causal effect of the mediator on the outcome (beta2). The formula utilized for calculating the mediating effect is: Mediating Effect=beta1×beta2 19. The steps of the mediation analysis were shown in Figure 2.

## 3. Results

### 3.1 Selection of exposure variable: bidirectional MR analysis between GM and HCC

**Figure 3:**
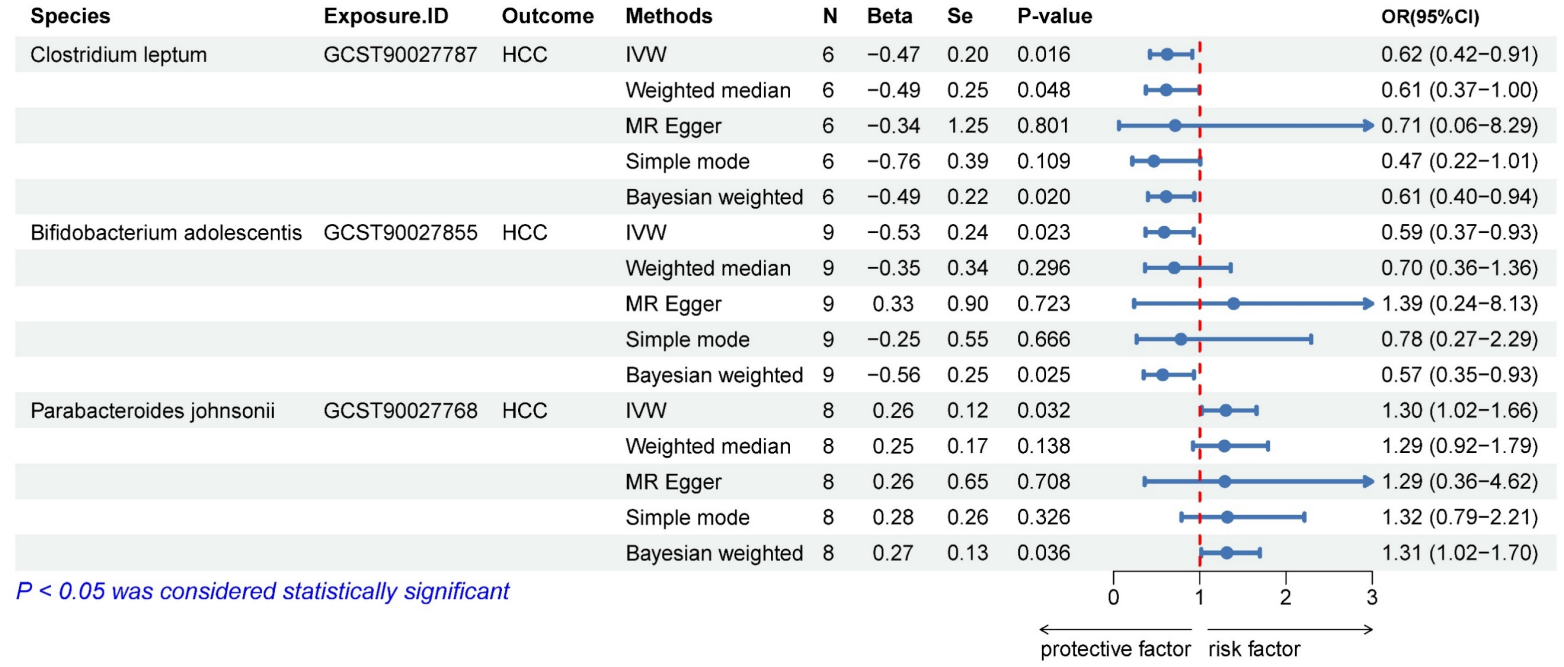
Causal relationship between GM and HCC. N: the number of SNPs; Se: standard error.

We excluded SNPs with LD, those with palindrome structures, and those already associated with the pathway. Detailed information on instrumental variables is provided in the Figure S1. The IVW and BW methods indicated a causal association of *Clostridium leptum* (IVW, odds ratio [OR]: 0.62, 95% confidence interval [CI]: 0.42-0.91, p = 0.016), *Bifidobacterium adolescentis* (IVW, OR: 0.59, 95% CI: 0.37-0.93, p = 0.023), and *Parabacteroides johnsonii* (IVW, OR: 1.30, 95% CI: 1.02-1.66, p = 0.032) with HCC (Figure 3). The WM method yielded consistent conclusions in the causal association analysis of *C. leptum* (OR: 0.61, 95% CI: 0.37-1.00, p = 0.048) with HCC. However, the results of WM with *B. adolescentis* (p = 0.296) and *P. johnsonii* (p = 0.138) were negative, leading to their exclusion (Figure 3). Reverse MR analysis indicated no reverse causal relationship between *C. leptum* and HCC (OR: 0.99, 95% CI: 0.95-1.03, p = 0.658; Figure S2). Finally, *C. leptum* was selected as the exposure variable (*SNP1*).

### 3.2 Selection of mediator variable

#### 3.2.1 Bidirectional MR analysis between C. leptum and metabolites

We conducted MR analysis on SNPs representing 1400 metabolites in the CLSA cohort, identifying three metabolites with a causal relationship with *C. leptum* (Figure S3). These are phosphoethanolamine (PE; IVW, OR: 0.91, 95% CI: 0.83-0.99, p = 0.028), sphingomyelin (IVW, OR: 0.91, 95% CI: 0.85-0.99, p = 0.022), and X-12729 (IVW, OR: 1.12, 95% CI: 1.03-1.23, p = 0.012). However, the results of the WM did not support a causal effect for sphingomyelin (p = 0.082) and X-12729 (p = 0.080), leading to their exclusion. Reverse MR analysis suggested the absence of a reverse causal relationship between *C. leptum* and PE (OR: 1.04, 95% CI: 0.84-1.28, p = 0.705; Figure S2). Finally, PE was selected as the *SNP2*.

#### 3.2.2 Bidirectional MR analysis between PE and HCC

The IVW method showed that PE was negatively associated with the risk of HCC (OR: 0.71, 95% CI: 0.541-0.935, p = 0.015; Figure S3). Reverse MR analysis indicated no reverse causal relationship between them (OR: 0.99, 95% CI: 0.98-1.01, p = 0.322; Figure S2). Therefore, PE was chosen as the mediator variable.

### 3.3 Results of mediation analysis

**Figure 4:**
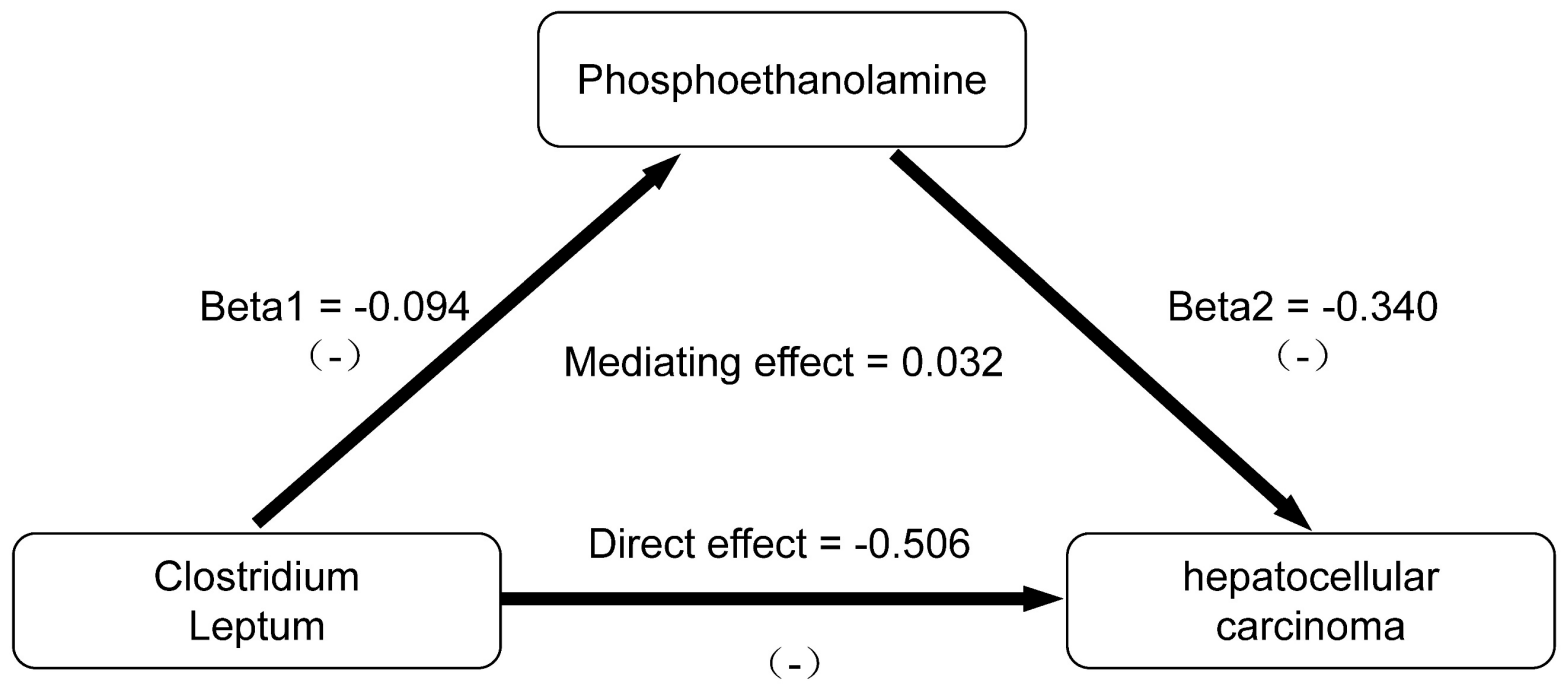
Results of mediation analysis.

We conducted an analysis of PE as a mediator of the pathway from *C. leptum* to HCC. The effect values were derived from the Beta coefficients of the IVW method, as depicted in Figure 4. Through two-step MR analysis, we calculated a potential mediation effect of PE levels (proportion mediated = -6.725%, 95% CI = 12.96% to -26.41%) in the causal association between *C. leptum* and the risk of HCC. This finding suggests that an increase of one standard deviation in *C. leptum* abundance would result in a 38% decrease in the risk of HCC (OR: 0.62 [95% CI, 0.42-0.91]), accompanied by a 9% decrease in PE levels (OR: 0.91 [95% CI: 0.84-0.99]). Simultaneously, an increase in PE levels by one standard deviation is associated with a 29% decrease in the risk of HCC (OR: 0.71 [95% CI: 0.54-0.94]). Reverse MR analysis indicated the absence of a reverse causal relationship. We observed that PE plays a role in negative feedback regulation in the gut-liver axis regulation process. When the abundance of GM increases, PE reduces this protective effect. Conversely, when the abundance of GM decreases, an increase in PE weakens the negative effects of GM instability.

### 3.4 Results of sensitivity analysis and visualization

The F-statistics for the selected SNPs are all above 10 (Figure S1), indicating a low likelihood of weak instrument bias for these instrumental variables. The r^2^ values ranging from 0 to 1 indicated that the two SNPs are randomly distributed, with no LD (Figure S1). Supplementary Table S1 presents the results of three sensitivity analyses. The p-values for Cochran's Q were all >0.05, the intercepts of Egger regression were close to zero, and the p-values were all >0.05. After removing outlier SNPs with MR-PRESSO, the p-values were all >0.05. These results demonstrate that our analysis adheres to the three fundamental assumptions of MR. We also visualized the results of the MR analysis. Scatter plots illustrated the trends of effects obtained from different parameter estimation methods (Figure S4). Leave-one-out analysis assessed the impact of each SNP on the overall causal estimate (Figure S5). Funnel plots indicated the results of heterogeneity assessment using IVW and MR-Egger (Figure S6). Forest plots illustrated the strength of the association between each SNP and the outcome (Figure S7).

## 4. Discussion

This study utilized the latest GWAS database to identify potential causal relationships between the gene expression of three GM and HCC. Additionally, a two-step bidirectional MR analysis revealed that the potential link between gut microbiota and HCC risk might be mediated by phosphoethanolamine levels (proportion mediated = -6.725%). Our findings indicate that increased gene expression abundance of *C. leptum* could reduce the risk of HCC. Furthermore, the protective effect of this GM was regulated by the levels of PE through negative feedback modulation. To the best of our knowledge, this is the first study reporting causal relationships involving plasma metabolites mediating interactions between GM and HCC.

Our study revealed that an increase in *C. leptum* abundance is associated with a reduced risk of HCC. Ma *et al.* conducted a MR analysis between GM and HCC in 2022. However, they did not perform a bidirectional analysis. They identified F_Ruminococcaceae and G_Porphyromonadaceae as protective factors for liver cancer 7, originating from the family and genus within bacterial classification. Unfortunately, they did not specify the species within the genus to which these bacteria belong. By contrast, our study utilized the latest published GWAS database to identify three different species of gut microbes that have a causal relationship with HCC. Another research of MR analysis was published by Jiang *et al.* But the exposure data were from the Western populations, whilst the outcome data were from the East Asians population. Populations from two independent samples of the different ethnic background could result in the winner's curse bias 7, 8. In our study, all databases were sourced from Western populations, effectively overcoming the winner's curse bias caused by sample overlap.

The bidirectional liver-gut axis, composed of the portal and biliary systems, serves as the anatomical foundation for the interplay between GM and liver diseases 32. On the one hand, nutrients, GM, and their metabolic products enter the liver through the portal vein system. On the other hand, various substances produced by the liver can enter the intestine through the biliary system, thereby influencing the intestinal microenvironment. Ren *et al.* 33 characterized the gut microbiome in 75 early HCC patients, 40 cirrhosis patients, and 75 healthy controls using 16S rRNA sequencing technology. They found that the abundance of GM profiles varies at different stages of diseases, revealing the correlation between gut microbial communities and liver diseases.

Our study demonstrates a causal relationship between gut microbiota composition and hepatocellular carcinoma. Previous research has also confirmed a significant correlation between gut microbiota and the risk of HCC. This is consistent with our study findings. Research suggests that GM, through various mechanisms such as altering intestinal barrier permeability, regulating inflammatory factors, endotoxins, and modulating the immune microenvironment, can directly or indirectly promote or inhibit the occurrence of tumors 34. Trautwein *et al.* found that the loss of *Akkermansia muciniphila* correlates with hepatic monocytic myeloid-derived suppressor cells abundance, and its reintroduction restores intestinal barrier function, strongly reducing liver inflammation and fibrosis, thereby inhibiting the development of liver cancer 35. In addition to shaping the inflammatory microenvironment, GM can also influence the immune barrier. Sharpe and colleagues found that a specific gut bacterium, *Coprobacillus cateniformis*, enhances the efficacy of PD-1 checkpoint blockade therapy by downregulating the expression and activity of immune molecules PD-L2 and repulsive guidance molecule b 36. Balancing and optimizing the GM could have a significant impact on controlling the development of tumors.

In our study, mediation MR analysis revealed that *C. leptum* is a protective bacterium against HCC and is negatively regulated by PE. Leptum is one of the major taxonomic groups within the *Clostridium* genus. In a study characterizing the dominant GM in obese patients with non-alcoholic fatty liver disease, the quantity of *Faecalibacterium prausnitzii* colonies was significantly lower in the obesity with non-alcoholic fatty liver disease group than in the simple obesity group. Additionally, *F. prausnitzii* is positively impacted by *C. leptum* 37, suggesting that Leptum may be a protective bacterial group, consistent with our research findings. Du and colleagues discovered that a significant enrichment of the Clostridia class can enhance antigen presentation and effector T cell function through the cGAS-STING-IFN-I pathway, thereby boosting anti-tumor immune responses 38. Simultaneously, Palamidi *et al.* found that adding organic acid supplements to food can increase the abundance of *C. leptum* subgroups (p = 0.040), thereby upregulating the expression of intestinal mucosal protective gene *MUC2* and improving intestinal barrier protection 39. These studies collectively indicate that *C. leptum*, as a protective bacterial group, can influence the occurrence and development of HCC through various mechanisms.

In mediation analysis, we discovered that PE, a metabolite, could mitigate the risk of HCC. Several studies have noted that levels of PE and its associated metabolites are influenced by the GM 40-43. Ferreira *et al.* previously reported that synthetic PE exhibited anticancer effects in Ehrlich ascites carcinoma by inducing apoptosis 44. Additionally, synthetic PE was found to induce cell cycle arrest and apoptosis in breast cancer cells through the mitochondrial pathway 45. These findings suggest that PE may act as a protective factor, aligning with the results of our MR analysis. A study on non-Hodgkin lymphoma indicated that the enzyme phospholipase-C could hydrolyze phosphatidylethanolamine and phosphatidylcholine into PE and diacylglycerol, all known to be involved in apoptosis via cell cycle signaling 46. Chemical reactions, signaling pathways, and various metabolic intermediates are interconnected in the human body. Understanding the causal role of metabolites in disease etiology can offer manageable intervention points for treatment.

MR is a potent causal inference method that can identify genetic variants associated with the study target. It overcomes the inherent limitations of traditional observational studies and diminishes the impact of confounding factors and reverse causation on the results 47. However, our study still has some limitations. First, despite employing various algorithms to mitigate confounding factors, SNPs may still be influenced by potential horizontal pleiotropy. Second, our GWAS data exclusively originated from Western countries. These results require validation in other ethnic groups owing to genetic, environmental, and lifestyle differences between Western and Eastern populations. Third, although our results indicated that certain GM and metabolites serve as protective factors for HCC, further mechanistic studies and randomized controlled trials are necessary to validate these findings. Since the findings from MR analysis rely solely on genetic evidence, additional mediators besides PE may also contribute to the interaction between the GM and HCC. Further research is warranted to explore these substances for a more comprehensive understanding of the complex interplay between GM and hepatocellular carcinoma.

## 5. Conclusions

This bidirectional mediation MR analysis suggests that PE may mediate the causal relationship between GM and HCC. Targeting these gut microbes could offer potential avenues for the prevention and treatment of HCC. However, further investigation is warranted to elucidate the underlying mechanisms linking GM and liver cancer.

## Supplementary Material

Supplementary figures and table.

## Figures and Tables

**Table 1:** Data sources and information used in this study.

Vairables	ID	Data sources	Year	Race	SNPs	Participants	Web resource
Gut microbiota	PMID: 35115690	Dutch Microbiome Project	2022	Northern Netherlands	-	7,738	https://www.ebi.ac.uk/gwas/publications/35115690
Metabolite	PMID: 36635386	Canadian Longitudinal Study on Aging	2023	Canadian	-	8,299	https://www.ebi.ac.uk/gwas/publications/36635386
HCC	finngen_R10_C3_HEPATOCELLU	FinnGen Consortium	2023	European	21, 303, 829	314,193	https://storage.googleapis.com/finngen-public-data-r10/summary_stats/finngen_R10_C3_HEPATOCELLU_CARC_EXALLC.gz

SNPs: single nucleotide polymorphisms; HCC: hepatocellular carcinoma

## Abbreviations

GM: gut microbiota
HCC: hepatocellular carcinoma
GWAS: genome-wide association studies
IVW: Inverse Variance Weighted
OR: Odds ratio
CI: confidence interval
PE: Phosphoethanolamine
CXCL16: chemokine ligand 16
NKT cell: natural killer T cell
MR: Mendelian randomization
IV: instrumental variable
MA: mediation analysis
SNP: single nucleotide polymorphism
LD: linkage disequilibrium
HBV: hepatitis B virus
SE: standard error
WM: Weighted Median
IVW: Inverse Variance Weighted
BW: Bayesian weighted
MR-PRESSO: Mendelian randomization pleiotropy residual sum and outlier
DMP: Dutch Microbiome Project
CLSA: Canadian Longitudinal Study on Aging
RNA: Ribonucleic Acid
PD-1: programmed death 1
PD-L2: programmed cell death ligand 2
RGMb: repulsive guidance molecule b
NAFLD: non-alcoholic fatty liver disease
