# Supplementary material for: Exploring the Mediation Effect of Metabolite Levels on the Association Between Gut Microbiota and HCC: A two-step, two-sample bidirectional Mendelian Randomization
Source: J Cancer. 2024 May 30;15(12):3975–83. doi: 10.7150/jca.96579 (PMC11190768; doi:10.7150/jca.96579)
Supplement: Supplementary file 1 — Supplementary figures and table. [file jcav15p3975s1.pdf]

SNP	N	Exposure	Outcome	EA	OA	Beta	Se	P-value	OR(95%CI)	r2	F
rs11858399	6	CL	HCC	T	C	−0.37	0.46	0.42	0.69 (0.28−1.69)	0.0029	22.71
rs17398712				C	G	−0.80	0.47	0.09	0.45 (0.18−1.14)	0.0029	22.47
rs67194754				G	A	0.39	0.53	0.47	1.48 (0.52−4.21)	0.0027	20.66
rs7073292				G	A	−1.00	0.47	0.04	0.37 (0.15−0.93)	0.0030	23.25
rs72662817				G	A	−0.79	0.48	0.10	0.46 (0.18−1.17)	0.0025	19.54
rs79201749				A	G	−0.19	0.42	0.65	0.83 (0.36−1.89)	0.0028	21.46
rs11858399	7	CL	PE	T	C	−0.14	0.12	0.25	0.87 (0.70−1.10)	0.0029	22.71
rs17398712				C	G	−0.19	0.11	0.08	0.83 (0.67−1.02)	0.0029	22.47
rs67194754				G	A	0.14	0.12	0.23	1.15 (0.91−1.46)	0.0027	20.66
rs7073292				G	A	−0.09	0.11	0.40	0.91 (0.73−1.13)	0.0030	23.25
rs72662817				G	A	−0.15	0.12	0.21	0.86 (0.68−1.09)	0.0025	19.54
rs79201749				A	G	−0.08	0.11	0.43	0.92 (0.75−1.13)	0.0028	21.46
rs9356977				A	C	−0.12	0.12	0.30	0.89 (0.70−1.11)	0.0028	21.38
rs11238055	24	PE	HCC	G	A	0.86	0.74	0.24	2.37 (0.56−10.07)	0.0026	21.33
rs113366355				T	C	−0.73	0.89	0.41	0.48 (0.08−2.75)	0.0025	20.35
rs116750842				A	C	−1.23	0.67	0.07	0.29 (0.08−1.10)	0.0033	27.11
rs117711183				C	G	−0.31	0.87	0.72	0.73 (0.13−4.06)	0.0028	22.88
rs11784095				A	C	1.13	0.95	0.24	3.09 (0.48−19.94)	0.0027	22.41
rs12364287				A	C	−1.32	0.86	0.13	0.27 (0.05−1.45)	0.0026	21.79
rs1256335				A	G	−0.02	0.68	0.98	0.98 (0.26−3.72)	0.0048	39.93
rs12757377				G	A	0.29	1.00	0.77	1.34 (0.19−9.56)	0.0025	20.41
rs13062174				G	A	−0.43	0.75	0.57	0.65 (0.15−2.85)	0.0036	30.02
rs140800109				T	A	−0.28	0.78	0.72	0.76 (0.16−3.49)	0.0029	24.09
rs142494088				A	G	−0.89	0.74	0.23	0.41 (0.10−1.75)	0.0024	19.95
rs149029212				T	C	−0.39	0.24	0.10	0.68 (0.43−1.08)	0.0024	20.23
rs154803				A	T	−1.20	0.80	0.14	0.30 (0.06−1.45)	0.0024	19.54
rs41291072				T	C	0.25	0.68	0.71	1.28 (0.34−4.90)	0.0031	25.65
rs567934844				C	A	0.44	0.86	0.61	1.55 (0.29−8.37)	0.0024	19.76
rs72987261				A	C	−0.27	0.67	0.69	0.76 (0.20−2.86)	0.0027	22.54
rs75129304				G	T	−1.84	1.00	0.07	0.16 (0.02−1.12)	0.0026	21.35
rs75129536				A	G	0.03	0.85	0.97	1.03 (0.19−5.45)	0.0024	19.85
rs76305989				C	A	−0.48	0.83	0.56	0.62 (0.12−3.18)	0.0028	22.80
rs77363810				C	T	0.58	1.50	0.70	1.79 (0.10−33.66)	0.0024	19.79
rs77399645				T	C	0.20	1.46	0.89	1.22 (0.07−21.31)	0.0027	22.30
rs7745468				C	A	−0.02	0.84	0.98	0.98 (0.19−5.05)	0.0028	23.38
rs78763495				A	C	−0.51	0.75	0.50	0.6 (0.14−2.62)	0.0028	22.79
rs78767165				A	G	−0.76	1.10	0.49	0.47 (0.05−40)	0.0024	20.11

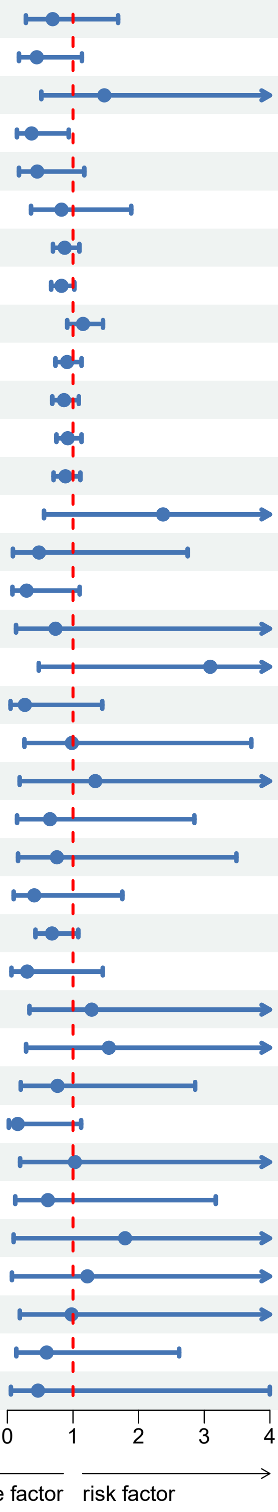

P < 0.05 was considered statistically significant

Supplementary Figure S1: IVs used in the MR analysis.

N: the number of SNPs; EA: effective allele; OA: other alleles; SE: standard error

Exposure	Outcome	Methods	N	Beta	Se	P-value	OR(95%CI)
HCC	CL	IVW	30	0.03	0.05	0.54	1.03 (0.94−1.12)
		Weighted median	30	<0.01	0.03	0.90	1.00 (0.94−1.05)
		MR Egger	30	−0.01	0.02	0.66	0.99 (0.95−1.03)
		Simple mode	30	0.05	0.05	0.34	1.05 (0.95−1.16)
PE	CL	IVW	12	−0.2	0.47	0.68	0.82 (0.33−2.06)
		Weighted median	12	−0.02	0.14	0.91	0.98 (0.74−1.30)
		MR Egger	12	0.04	0.11	0.71	1.04 (0.84−1.29)
		Simple mode	12	−0.07	0.24	0.78	0.93 (0.58−1.50)
HCC	PE	IVW	57	−0.01	0.02	0.70	0.99 (0.97−1.02)
		Weighted median	57	<0.01	0.01	0.75	1.00 (0.98−1.02)
		MR Egger	57	−0.01	0.01	0.32	0.99 (0.98−1.01)
		Simple mode	57	0.01	0.02	0.72	1.01 (0.97−1.05)

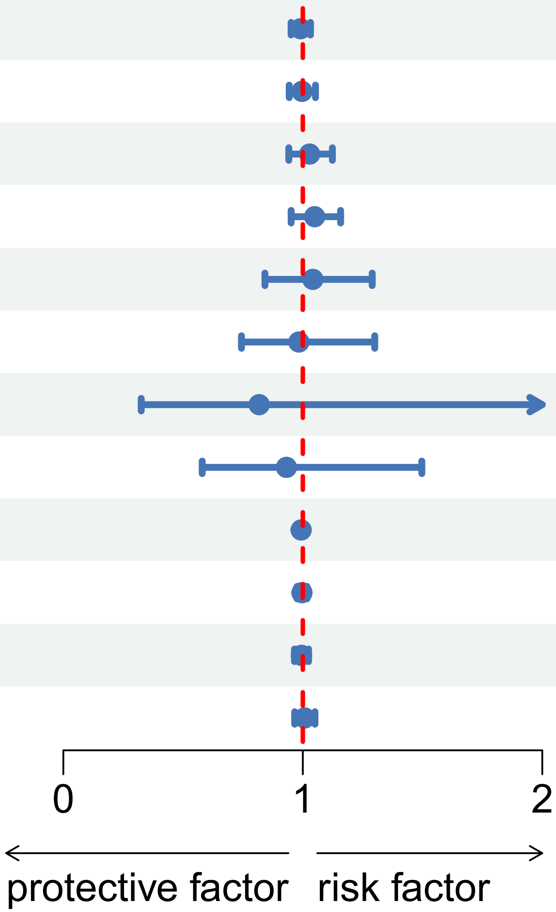

P < 0.05 was considered statistically significant

Supplementary Fig. S2: Results of reverse MR analysis.

N: the number of SNPs; SE: standard error; IVW: Inverse Variance Weighted

Exposure	Outcome	Methods	N	Beta	Se	P-value	OR(95%CI)
CL	PE	IVW	7	−0.09	0.04	0.028	0.91 (0.84−0.99)
		Weighted median	7	−0.12	0.06	0.037	0.89 (0.80−0.99)
		MR Egger	7	−0.29	0.24	0.274	0.75 (0.47−1.19)
		Simple mode	7	−0.12	0.08	0.173	0.88 (0.76−1.03)
		Bayesian weighted	7	−0.10	0.05	0.030	0.91 (0.83−0.99)
CL	Sphingomyelin	IVW	7	−0.09	0.04	0.020	0.91 (0.85−0.99)
		Weighted median	7	−0.08	0.05	0.082	0.92 (0.84−1.01)
		MR Egger	7	−0.16	0.22	0.483	0.85 (0.56−1.30)
		Simple mode	7	−0.11	0.07	0.148	0.89 (0.78−1.02)
		Bayesian weighted	7	−0.09	0.04	0.030	0.91 (0.84−0.99)
CL	X−12729	IVW	7	0.11	0.05	0.012	1.12 (1.03−1.23)
		Weighted median	7	0.11	0.06	0.080	1.12 (0.99−1.26)
		MR Egger	7	0.32	0.25	0.265	1.37 (0.84−2.25)
		Simple mode	7	0.05	0.09	0.627	1.05 (0.88−1.25)
		Bayesian weighted	7	0.12	0.05	0.020	1.12 (1.02−1.24)
PE	HCC	IVW	24	−0.34	0.14	0.015	0.70 (0.45−1.07)
		Weighted median	24	−0.37	0.23	0.107	0.69 (0.44−1.08)
		MR Egger	24	−0.36	0.22	0.112	0.71 (0.54−0.94)
		Simple mode	24	−0.27	0.38	0.472	0.76 (0.36−1.59)
		Bayesian weighted	24	−0.36	0.18	0.041	0.70 (0.49−0.98)

P < 0.05 was considered statistically significant

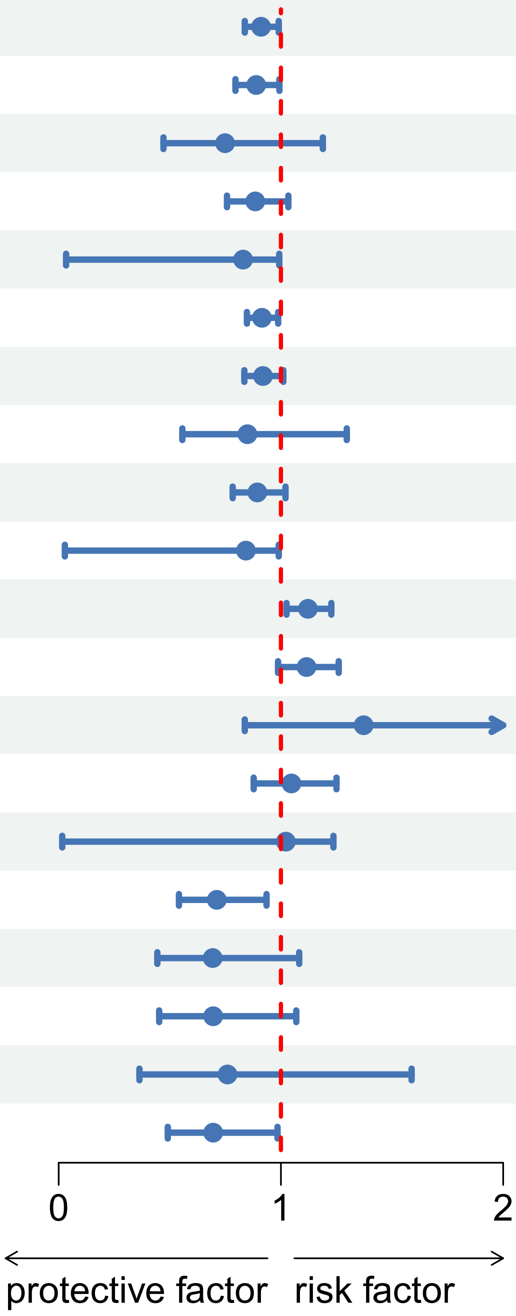

Supplementary Fig. S3: Main results of the MR analysis.
N: the number of SNPs; SE: standard error; IVW: Inverse Variance Weighted

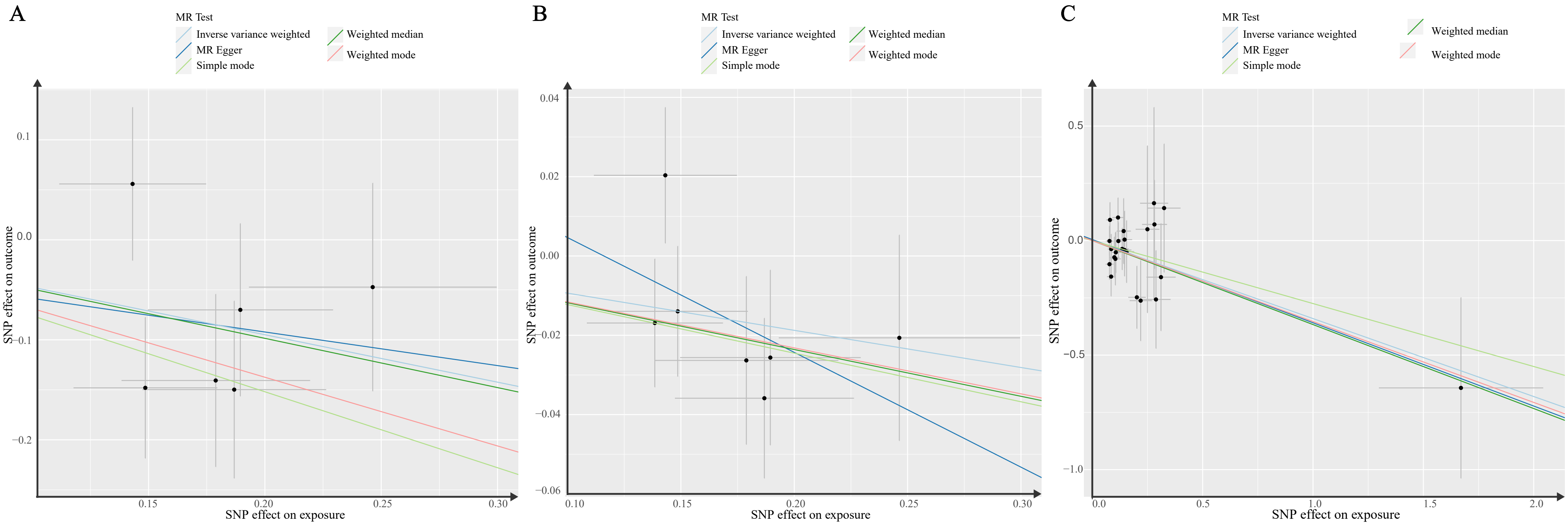

Supplementary Figure S4: Scatter plots. The y-axis represents the causal effect of IVs on the outcome, while the x-axis represents the effect on the exposure. The slope illustrates the effect of exposure on the outcome. (A)illustrates the trends of effects between CL and HCC. (B)illustrates the trends of effects between CL and PE. (C)illustrates the trends of effects between PE and HCC.

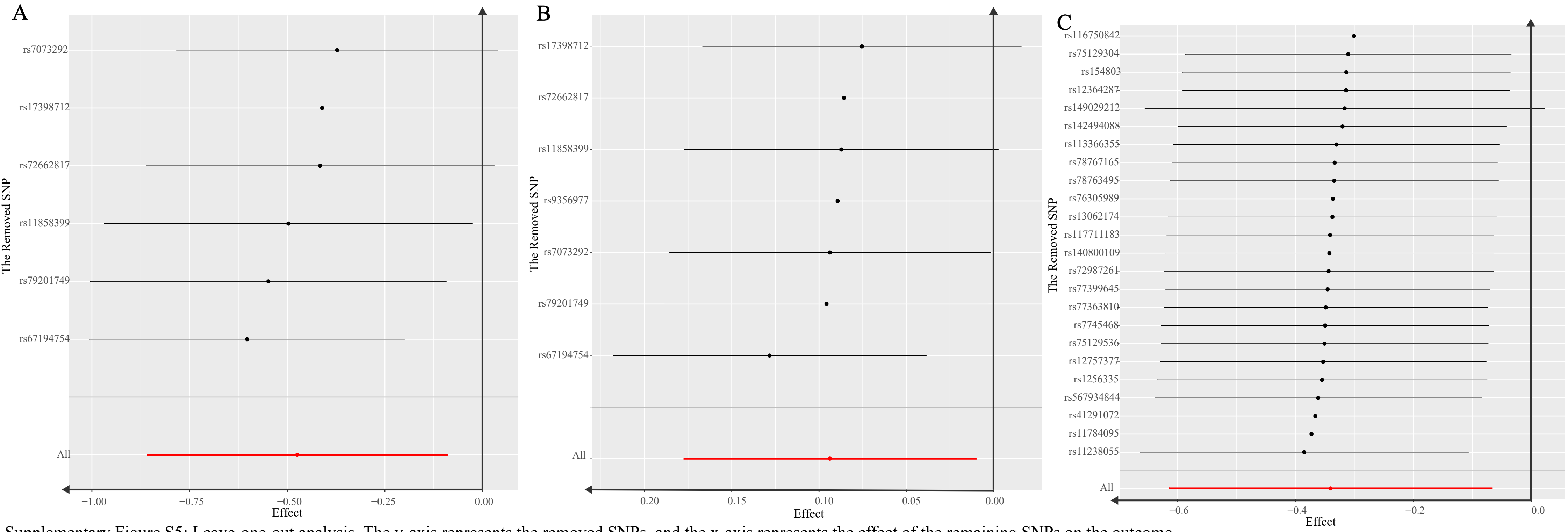

Supplementary Figure S5: Leave-one-out analysis. The y-axis represents the removed SNPs, and the x-axis represents the effect of the remaining SNPs on the outcome. (A)illustrates the stability of SNPs between CL and HCC. (B)illustrates the stability of SNPs between CL and PE. (C)illustrates the stability of SNPs between PE and HCC.

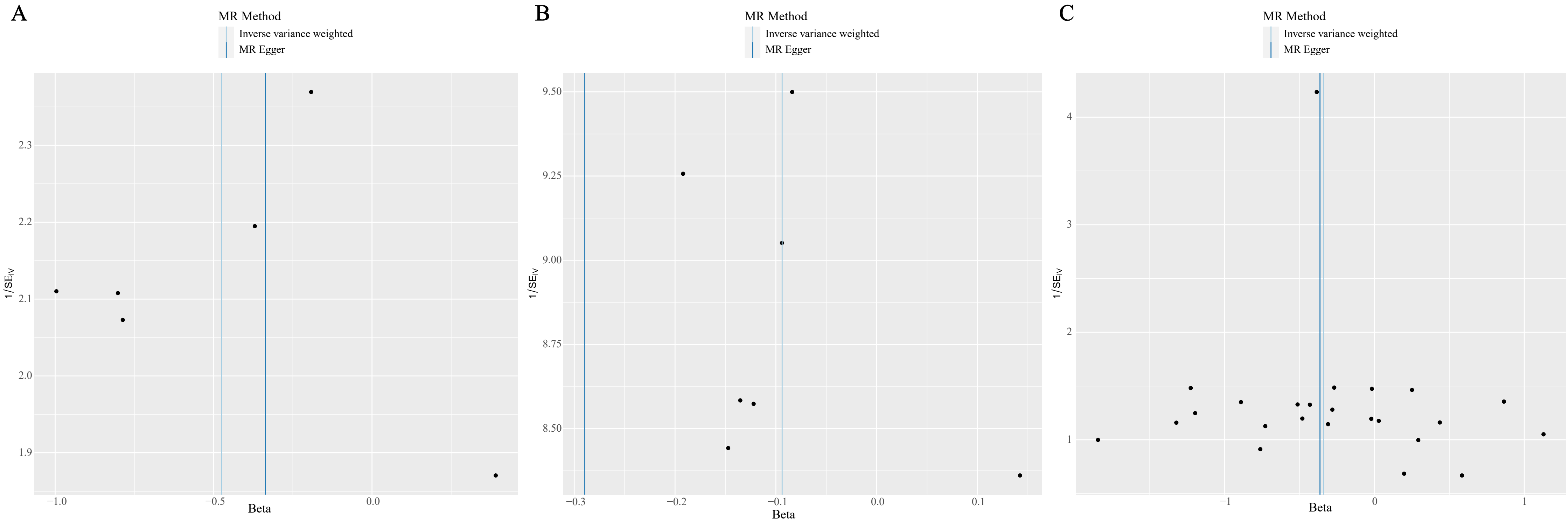

Supplementary Figure S6: Funnel plots. Symmetry indicates the absence of heterogeneity in IVs.
(A)illustrates the heterogeneity of SNPs between CL and HCC. (B)illustrates the heterogeneity of SNPs between CL and PE. (C)illustrates the heterogeneity of SNPs between PE and HCC.

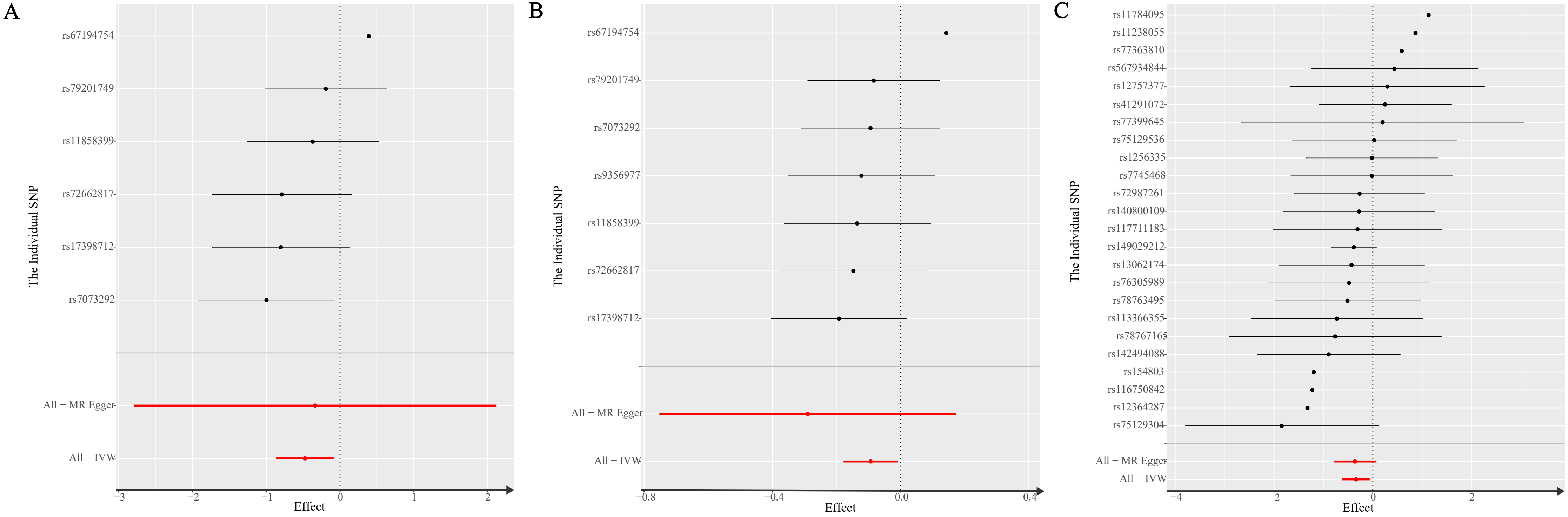

Supplementary Figure S7: Forest plots. The y-axis represents individual SNPs, while the x-axis represents the effect of each SNP on exposure.
(A)illustrates the association between each SNP and the outcome between CL and HCC. (B)illustrates the association between each SNP and the outcome between CL and PE. (C)illustrates the association between each SNP and the outcome between PE and HCC.

Table S1 The results of sensitivity analysis.

Exposure	Outcome	Cochran’s Q			Egger regression		MR-PRESSO
		Q	Q_df	Q_pval	Intercept	P-value	P-value
CL	HCC	5	5	0.389	-0.02	0.916	0.550
CL	PE	5	6	0.529	0.03	0.437	0.455
PE	HCC	16	23	0.874	0.87	0.893	0.878

Cochran's Q test was utilized to examine heterogeneity. Egger regression and MR-PRESSO were used to identified horizontal pleiotropic

CL: *C. leptum*; PE: phosphoethanolamine; HCC: hepatocellular carcinoma;
